# Microfluidic Chip with Two-Stage Isothermal Amplification Method for Highly Sensitive Parallel Detection of SARS-CoV-2 and Measles Virus

**DOI:** 10.3390/mi12121582

**Published:** 2021-12-19

**Authors:** Qin Huang, Xiaohui Shan, Ranran Cao, Xiangyu Jin, Xue Lin, Qiurong He, Yulei Zhu, Rongxin Fu, Wenli Du, Wenqi Lv, Ying Xia, Guoliang Huang

**Affiliations:** 1 Department of Biomedical Engineering, School of Medicine, Tsinghua University, Beijing 100084, China; xiaoqingzi12345@163.com (Q.H.); 17763384805@163.com (X.S.); 18810913852@163.com (X.J.); ruixuezhaofn@yeah.net (X.L.); thu_frx@tsinghua.edu.cn (R.F.); wenlidu1230@163.com (W.D.); 18254369766@163.com (W.L.); 2 Sichuan Center for Disease Control and Prevention, Chengdu 610041, China; ranran.cao@foxmail.com; 3 West China School of Public Health and West China Fourth Hospital, Sichuan University, Chengdu 610041, China; heqiurong2009@163.com (Q.H.); yulei_zhu@yeah.net (Y.Z.)

**Keywords:** two-stage isothermal amplification, microfluidic chip, parallel molecular diagnostic, SARS-CoV-2, measles virus

## Abstract

A two-stage isothermal amplification method, which consists of a first-stage basic recombinase polymerase amplification (RPA) and a second-stage fluorescence loop-mediated isothermal amplification (LAMP), as well as a microfluidic-chip-based portable system, were developed in this study; these enabled parallel detection of multiplex targets in real time in around one hour, with high sensitivity and specificity, without cross-contamination. The consumption of the sample and the reagent was 2.1 μL and 10.6 μL per reaction for RPA and LAMP, respectively. The lowest detection limit (LOD) was about 10 copies. The clinical amplification of about 40 nasopharyngeal swab samples, containing 17 SARS-CoV-2 (severe acute respiratory syndrome coronavirus) and 23 measles viruses (MV), were parallel tested by using the microfluidic chip. Both clinical specificity and sensitivity were 100% for MV, and the clinical specificity and sensitivity were 94.12% and 95.83% for SARS-CoV-2, respectively. This two-stage isothermal amplification method based on the microfluidic chip format offers a convenient, clinically parallel molecular diagnostic method, which can identify different nucleic acid samples simultaneously and in a timely manner, and with a low cost of the reaction reagent. It is especially suitable for resource-limited areas and point-of-care testing (POCT).

## 1. Introduction

The recognition of infection pathogens is crucial for the assessment of disease state and treatment efficacy; therefore, diagnosing infectious diseases accurately and in a timely manner is important, especially for diseases caused by multiple pathogens [1,2,3]. For example, pneumonia can be caused by a variety of organisms, including bacteria, bacteria-like organisms, viruses and fungi [4,5]. Antibiotics are usually used to treat bacterial pneumonia, but inappropriate prescribing of antibiotics (for example, for viral infections) may result in drug resistance [6]. However, it takes time to identify the type of pathogen causing pneumonia and to choose the best antibiotic to treat it. The most common method to confirm an infection is a blood test [7], which can identify the type of organism. However, it is impossible for precise identification, such as telling which kind of virus or bacteria caused the infection. Culture-based microbiological tests, which are verified as the golden standard method for accurate pathogen identification, have suffered from a delay between sampling and result reporting, ranging from hours to days; furthermore, this method also requires a skilled experimenter and a facilitated laboratory [8].

Although molecular diagnostic methods, especially PCR, have developed rapidly throughout the last several decades [9], the clinical application of these methods was still relatively limited until the year 2020. Since the emergence of a respiratory disease caused by a new kind of coronavirus, SARS-CoV-2, there have been more than 229 million confirmed cases of COVID-19 [10]. Due to the fact that molecular diagnostic methods can detect a large number of pathogens within a greatly shortened time (usually 2 to 3 h) with accuracy, molecular diagnostics have become the gold standard methods for SARS-CoV-2 testing, particularly the nucleic acid test method [11]. However, the test is only available in fully equipped first-level hospitals. The application in community clinics and clinics in villages and towns, especially in resource-limited areas and countries, is still hampered because the thermal cycling (PCR) process complicates the instrumentation; this means a high cost of equipment and a need for professional molecular experimenters. Moreover, most of the existing nucleic acid molecular diagnostic assays can only detect one single target in a test, which is limited in scope. Although multiplex PCR (mPCR) has the ability to amplify many nucleic acid (NA) targets in a single reaction [12,13], it requires probes with different color fluorophores for multiple detection, which complicates the assay and increases the cost [14].

In recent years, isothermal amplification methods with no need for a temperature controlling component, such as loop-mediated isothermal amplification (LAMP) [15], recombinase polymerase amplification (RPA) [16], and nucleic acid sequence-based amplification (NASBA) [17], have attracted interest among researchers [18,19,20,21]. A few groups have developed multiplexed RPA [22,23] and multiplexed LAMP assays [24,25,26,27] for co-detection of a small number (i.e., ≤4) of targets. There are also some isothermal amplification methods based on microfluidic chips that have been developed for rapid detection and identification of pathogens [28,29,30,31].

Specifically, research based on isothermal amplification methods have been reported for the detection of SARS-CoV-2. As reported by Kim Y. et al. (Preprint), they reported a single-strand RPA for visual detection of SARS-CoV-2 [32], while Zhang Y. developed a AuNP-based colorimetric visual assay, which is called Cas12a-assisted RT-LAMP/AuNP (CLAP), for on-site diagnosis of COVID-19 [33]. El-Tholoth M. also used a two-stage isothermal amplification (Penn-RAMP), which was developed by Song J. in 2017 [34] for the detection of SARS-CoV-2 [35]. However, these methods were performed in tubes, which means large volume (25 to 50 µL) and the detection of only one target in a single tube.

To enhance the limits of detection, improve specificity, and enable parallel detection of multiple nucleic acid targets, we proposed a two-stage isothermal amplification, with an integrated microfluidic chip for the detection of different types of pathogens in a closed system, with a volume of 1 µL RPA, plus 10 µL LAMP for each target, during about one hour process; this consists of first-stage basic RPA and second-stage real-time LAMP, while products of RPA serve as templates for LAMP. The performance of the two-stage isothermal method was evaluated by comparing the limit of detection and reproducibility with basic RPA and fluorescence LAMP. The microfluidic chip’s capability was demonstrated by comparing the sensitivity and reproducibility of two-stage isothermal reactions on the microfluidic chip with in tubes. The ability to perform parallel detection of different types of targets on one chip was examined by detecting 40 clinical samples and comparing the results with traditional microbiological methods. The results led us to believe that the closed microfluidic chip system with the two-stage isothermal amplification method has high sensitivity, specificity and reproducibility, and consumes small volume reaction components with a fast reaction speed, which is suitable for real-time multiple pathogen diagnosis in a single detection run.

## 2. Materials and Methods

### 2.1. Targets and Sample Preparation 

Two viruses were targeted in this study: (1) SARS-CoV-2 (severe acute respiratory syndrome coronavirus), which is a pathogen that caused the outbreak of coronavirus disease 2019 (COVID-19) worldwide, and (2) Measles Virus (MV), which is an enveloped negative-strand RNA virus that causes measles and is highly contagious. Infected patients of both of these pathogens develop high fever and cold-like symptoms at an early stage, and can experience pneumonia or even death if the patient is elderly, very young, or immunocompromised. 

To detect and distinguish these two viruses from different infectious diseases, we used two different types of samples: (1) Plasmid DNA containing the targeted sequences, which were synthesized at Sangon Biotech (Shanghai, China) to mimic the SARS-CoV-2 and MV target, (2) clinical nasopharyngeal swab samples from SARS-CoV-2 and MV infected patients. Purified MV RNA and 40 nasopharyngeal swab samples from patients infected by COVID-19 or MV were obtained from the Center for Disease Control and Prevention (Sichuan, China). All clinical samples were heated at 98 °C for 15 min before nucleic acid extraction, according to biosafety laboratory requirements. RNA nucleic acid from Clinical samples was extracted using the Maxwell 16 Viral Total Nucleic Acid Purification Kit (Promega Co., Madison, WI, USA) at a Biological safety protection third-level laboratory (P3). The prepared nucleic acids were immediately stored at −20 °C until use.

### 2.2. RPA and LAMP Reaction in Tube

We performed the recombinase polymerase amplification (RPA) reaction for the first-stage amplification with the TwistAmp^®^ Basic Kit and TwistAmp^®^ Exo Kit (TwistDx Inc., Cambridge, UK). Targeting RNA experiments were conducted by adding 0.5 U/μL Avian Myeloblastosis Virus Reverse Transcriptase (AMV RT) (Promega Co., Madison, WI, USA) to synthesize a cDNA at first. The second-stage amplification of the loop-mediated isothermal amplification (LAMP) reaction was performed with a WarmStart LAMP Kit (New England BioLabs Inc., Beijing, China). The product of the RPA reaction was purified with a Universal DNA Purification Kit (Tiangen, Beijing, China).

The sequences for the primers are listed in Table 1. The RPA assays required a set of two primers for each target. The RPA primers were designed according to TwistAmp^®^ DNA amplification kits Combined instruction manual. LAMP assays required a set of six primers to amplify a specific target, including two outer primers (F3 and B3), two inner primers (forward inner primer (FIP) and backward inner primer (BIP)), and two loop primers (forward loop primer (LF) and backward loop primer (LB)). All LAMP primers were designed on the official primer design website (https://primerexplorer.jp/elamp4.0.0/index.html), and their specificities were verified by Basic Local Alignment Search Tool (BLAST) analysis (Version 4, Bethesda, MD, USA). All primers (as listed in Table 1) were ordered through Sangon Biotech (Shanghai, China).

The RPA and LAMP reactions in tubes were performed by using a real-time thermos-cycler (CFX96, Bio-Rad, Hercules, CA, USA). The first-stage RPA reaction was set at 39 °C for 15 min, then 1 μL aliquots of the RPA product were used as templates for the second-stage LAMP reaction, which was set at 65 °C for 50 min. The amplification of LAMP was monitored in real time by detecting its fluorescence signal.

### 2.3. Air-Insulated Microfluidic Chip Design 

**Figure 1:**
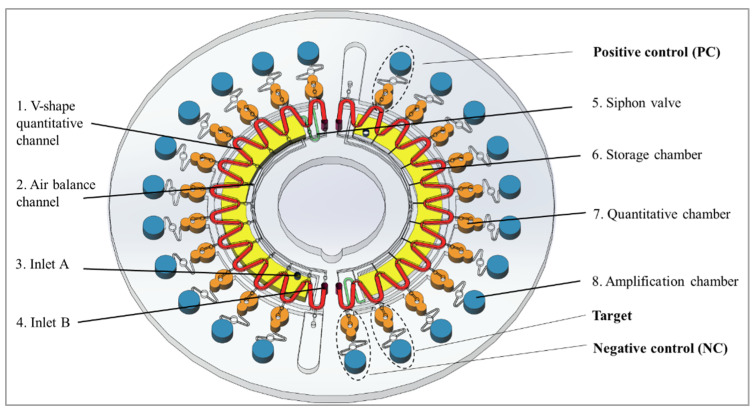
Three dimensional structure of the microfluidic chip.

Our air-insulated, disc-type microfluidic chip had a diameter of 62 mm and a thickness of 3.0 mm. The chip was designed with AutoCAD 2017. The chip was made of polymethyl methacrylate (PMMA) and fabricated by computer-controlled (CNC) milling. The structure of the chip is illustrated in Figure 1. It consisted of four layers: a basement layer carved with all the microstructures, two cover layers for encapsulation, and a film to seal the inlet holes. The basement layer consisted of two sides: side A was carved with microstructures for the RPA reaction, while side B was carved for the LAMP reaction. Side A consisted of a V-shape quantitative channel (width: 0.6 mm, depth: 0.4 mm), an air balance channel (width: 0.1 mm, depth: 0.1 mm), inlet A and inlet B. Side B consisted of a siphon valve (width: 0.3 mm, depth: 0.3 mm), a storage chamber (depth: 1.5 mm), a quantitative chamber (depth: 1.3 mm) and amplification chambers (depth: 1.5 mm). The V-shape channel was used to divide the RPA mix into quantitative chambers, with 2.10 μL of liquid distributed into each chamber. The quantitative chamber, with a volume of about 8.70 μL, was used for the RPA reaction and the quantification of the LAMP mix. The amplification chambers for the LAMP reactions had a diameter of 3.0 mm and a volume of 10.60 μL. The number of amplification chambers could be adjusted according to the number of targets and controls. The chip, illustrated in Figure 1, contained two identical parts, which could perform two samples on one chip for one detection. One hard polycarbonate (PC) film was used to cover side A, and one soft PC film was used to cover side B. Both PC films were tightly adhered to both sides of the chip to form an enclosed microfluidic chip.

### 2.4. RPA and LAMP Reactions on Microfluidic Chip

On the microfluidic chip, the reactions were performed with the same kits as in the tube, and with the same proportions of the components. After the basement layer was cleaned with 70% ethanol, the quantitative chamber was preloaded with RPA primers and Mg(OAc)_2_, and the amplification chamber was embedded with LAMP primers designed to identify one pathogen using low melting-point Sepharose CL-4B. The siphon valve was modified to be hydrophilic using the hydrophilic reagent (1% agarose in water). The chip was sealed with two PC films and compressed by a press machine to form an enclosed microfluidic chip. After that, the chip was stored at 4 °C until use.

**Figure 2:**
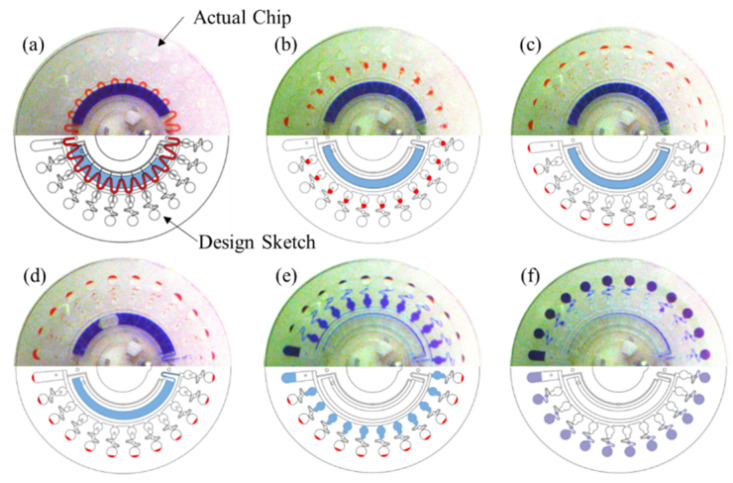
Illustration of the entire flow control of the chip. (**a**) The initial state of the chip with the RPA mix (red dye) and LAMP mix (blue dye), dried primers; (**b**) RPA mix was divided into quantitative chambers at 2000 rpm for 30 s; (**c**) the RPA product was transferred into amplification chamber as template for LAMP; (**d**) LAMP mix was primed into the siphon valve by the capillary action at 100 rpm for 30 s; (**e**) LAMP mix was transferred into the separated sub-volumes (10.6 μL per chamber) at 2000 rpm for 30 s; (**f**) LAMP mix was distributed into reaction chambers at 6000 rpm for 60 s.

For chip use, the RPA mix (25 μL) and LAMP mix (120 μL) were added separately into the V-shape channel and storage chamber using a pipette, through inlets A and B, respectively. After that, all of the inlets and outlets were sealed with adhesive tape. Figure 2 illustrates the entire flow control of the chip. Next we put the chip into the portable analyzer (Authorized patent ZL201110113608.1) which can conduct fully automate nucleic acid analysis. The entire flow control of the chip, on-chip RPA and LAMP reaction, and real-time fluorescent signal of LAMP amplification detection were all completed in the analyzer. The on-chip RPA was performed at 39 °C for 10 min, and on-chip LAMP was performed at 65 °C for 50 min.

## 3. Results

### 3.1. Comparison of Sensitivity and Reproducibility of Basic RPA Followed by Fluorescence LAMP (bRPA-LAMP) with Basic RPA and Fluorescence LAMP in Tube

To demonstrate the sensitivity of the bRPA-LAMP assay, we compared it with basic RPA and fluorescence LAMP in the tube. The targets were 1E+4, 1E+3, 1E+2, and 1E+1 copies of MV RNA. Three duplicate reactions were performed for each nucleic acid template concentration to evaluate reproducibility. The sensitivity of basic RPA was measured with agarose gel (1.5%) electrophoretograms of purified RPA products that were cleaned up with a DNA product purification kit (Tiangen, Beijing, China). The sensitivity of fluorescence LAMP and bRPA-LAMP were measured with the time of positive value (Tp), which was defined as the time at the second derivative inflexions of the exponential DNA amplification curves.

**Figure 3:**
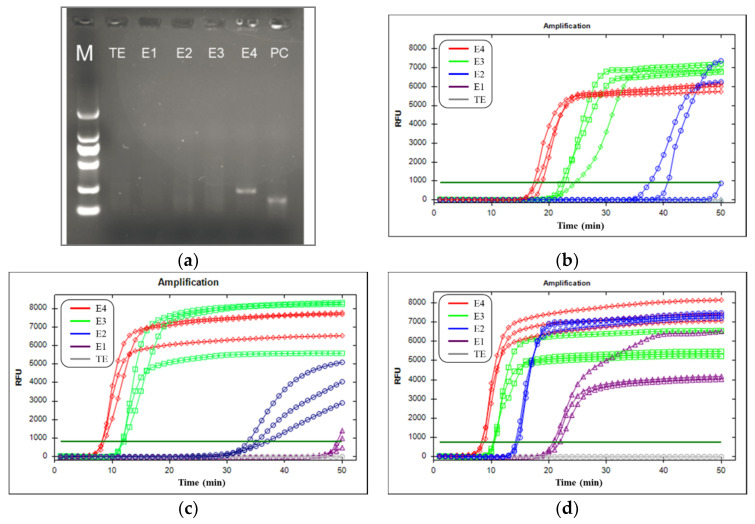
Comparison of sensitivity of bRPA-LAMP with basic RPA and fluorescence LAMP for MV RNA. (**a**) Agarose gel (1.5%) electrophoretogram image of purified basic RPA products; (**b**) real-time curves of LAMP; (**c**) real-time curves of unpurified RPA product followed by LAMP; (**d**) real-time curves of purified RPA product followed by LAMP. Note: PC: positive control.

As Figure 3 indicates, basic RPA measured by agarose gel (1.5%), electrophoretograms had the lowest sensitivity with a detection limit of 1E+4 copies of MV RNA. The LAMP process alone produced a detectable signal with 1E+3 copies, but had 2 positive signals out of three repeats for 1E+2 copies of MV RNA. In contrast, the bRPA-LAMP method yielded a significantly higher sensitivity than LAMP alone. The unpurified RPA product served as a template for fluorescence LAMP successfully producing a signal with samples containing 1E+2 copies of MV RNA. It is noteworthy that the purified RPA product that served as a template for fluorescence LAMP can detect samples containing 1E+1 copies of MV RNA as early as about 21.3 min. Its positive signal was produced even earlier than the LAMP with 1E+3 copies of MV RNA template. As Table 2 shows, the Tp values of RPA followed by fluorescence LAMP were significantly smaller than those of LAMP alone. The corresponding standard deviation (SD) values indicate that RPA followed by fluorescence LAMP, as well as LAMP, was reasonably reproducible (~4% relative standard deviation in the Tp values). Thus, we can see that the purified RPA product followed by LAMP had the highest sensitivity of 1E+1 copies, followed by the unpurified RPA product, followed by LAMP with a sensitivity of 1E+2 copies. Furthermore, LAMP had a sensitivity of 1E+3 copies, while basic RPA had the lowest sensitivity of 1E+4 copies.

### 3.2. Comparison of Sensitivity of bRPA-LAMP with LAMP on the Microfluidic Chip

We examined the performance of bRPA-LAMP on our microfluidic chip by comparing it with LAMP. MV was targeted by using its RNA at concentrations of 1E+3, 1E+2, and 1E+1 copies. There were 3 duplicate target bioreactors for bRPA-LAMP on one half of the microfluidic chip, and 3 duplicate target bioreactors for LAMP on the other half of the same chip. Reproducibility was evaluated by measuring the standard deviation of the Tp values of 3 repeats, as shown in Table 3.

**Figure 4:**
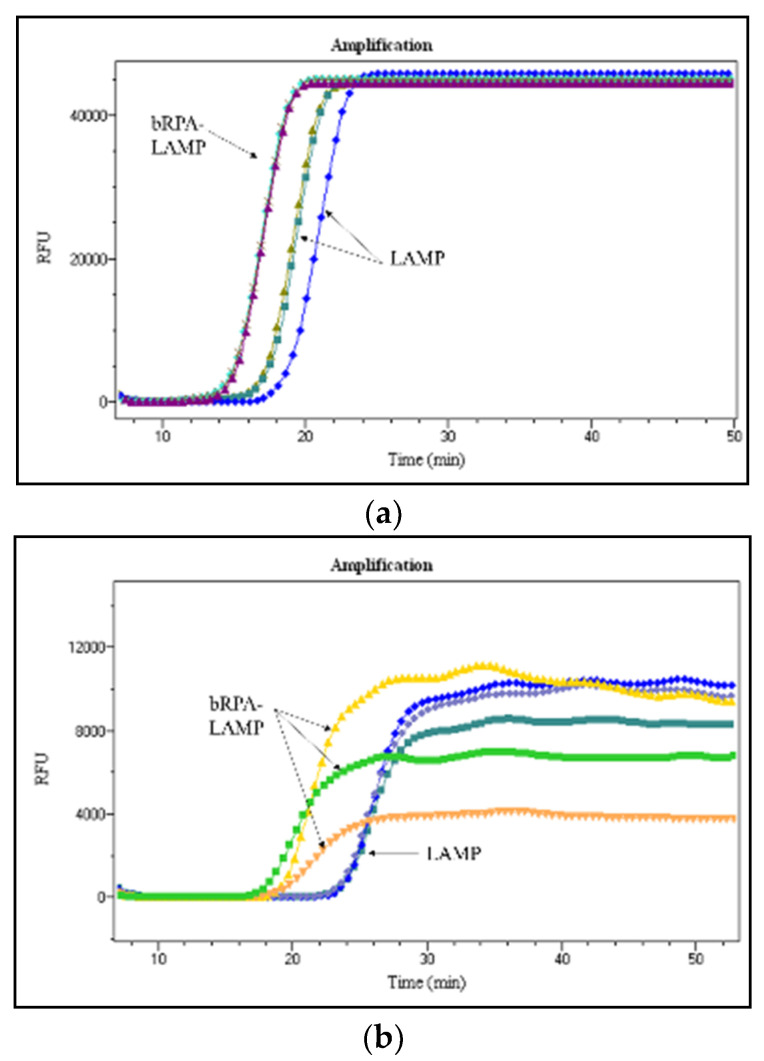

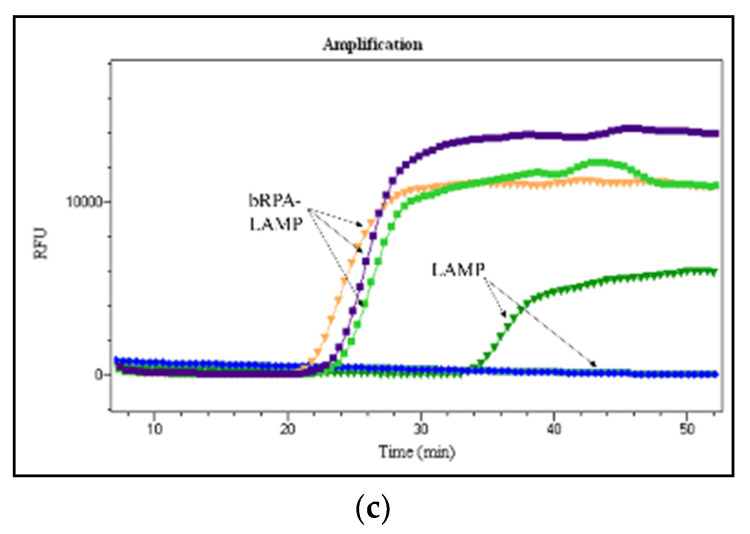
Comparison of sensitivity of bRPA-LAMP with LAMP on the microfluidic chip. (**a**) Real-time amplification curves for MV RNA at concentrations of 1E+3 copies; (**b**) real-time amplification curves for MV RNA at concentrations of 1E+2 copies; (**c**) real-time amplification curves for MV RNA at concentrations of 1E+1 copies.

Figure 4 depicts amplification curves with various concentrations of MV RNA. For the nucleic acid concentration at 1E+1 copies, bRPA-LAMP successfully detected the target at about 21 min, while LAMP only generated detectable signal for one chamber out of three duplicates. When the MV RNA concentration is 1E+2 copies, the Tp values are about 17.3 min and 21.8 min for bRPA-LAMP and LAMP, respectively. As for MV RNA at a concentration of 1E+3 copies, bRPA-LAMP produced signals about 3 min earlier than LAMP. For reproducibility, there was no big difference between bRPA-LAMP and LAMP, because they both had a SD of less than 0.5 min. 

When comparing the performance of bRPA-LAMP and LAMP on the microfluidic chip with the same reactions in the EP tubes, regardless of the fact that the reactions on the chip had a longer detection time for template concentration at 1E+3 copies, both methods had better performance for low template concentration (1E+1 and 1E+2 copies); this indicates higher sensitivity for low nucleic acid concentration samples on the microfluidic chip than in the tube.

### 3.3. Clinical Sensitivity and Specificity of Parallel Detection for Virus on the Same Microfluidic Chip

A total of 40 nasopharyngeal swab samples were classified by standard culture and PCR method, and were detected by using the parallel detection microfluidic chip. The 40 clinical samples included 23 MV positive samples, and 17 SARS-CoV-2 positive samples. Samples that contained one positive target were also used as negative samples for other targets. 

**Figure 5:**
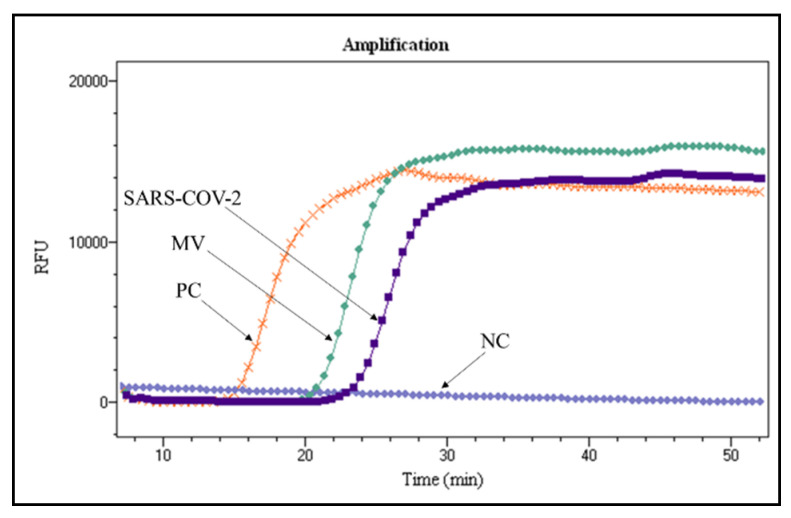
Real-time parallel detection for nasopharyngeal swab samples of SARS-CoV-2 and MV by using bRPA-LAMP on the same microfluidic chip. Note: PC: positive control; NC: negative control.

To test the parallel detection ability of the microfluidic chip, due to the possibility of multiple infection for respiratory diseases, we mixed nucleic acid extracted from these two kinds of clinical nasopharyngeal swab samples. Figure 5 indicates the result of parallel detection of SARS-CoV-2 and MV at the same time for the mixed nucleic acid sample. The Tp value for clinical samples is longer than that of plasmid DNA, because RNA needs some time to translate its RNA to cDNA.

To evaluate the clinical sensitivity and specificity of different targets, we also tested these samples separately. Table 4 shows that there was no cross-contamination between different targets on the microfluidic chip, with clinical specificity of 95.83% and 100% for SARS-CoV-2 and MV, respectively; this indicates the ability to perform parallel detection of multiple targets, by using one microfluidic chip and one test. Clinical sensitivity was 94.12% and 100% for SARS-CoV-2 and MV, respectively. We also obtained similar clinical sensitivity for SARS-CoV-2 by using the PCR method. The one SARS-CoV-2 positive sample that we failed to detect had a very low pathogenic concentration (<1E+1 copies). The fact that the microfluidic chip did not show a 100% clinical sensitivity for SARS-CoV-2 pointed the way of further improvement for our work, such as RPA product purification and nucleic Acid concentration.

## 4. Discussion

A series of work about microfluidic chips and parallel detection systems has been conducted by our group, including microfluidic chips made with different types of materials; with several designs of micro channels; and targeting bacteria that causes diseases and four types of Ebola virus species, by using separate microfluidic chips [36,37,38]. We also developed a portable nucleic acid analyzer (used in this study), which is even more suitable than ABI 7500 for trace samples, for the processing and detection of our corresponding microfluidic chips. The microfluidic chip-based isothermal detection system in this study was an integration and optimization of our previous research on microfluidic chips, while extending its clinical amplification extensively.

(1) Detecting different types of nucleic acid targets on one chip.

We made an effort to combine different types of nucleic acid (DNA and RNA) detection in one pot. This is convenient for the detection of clinical samples that may include bacteria and RNA viruses at the same time, such as nasopharyngeal swab samples. Correct classification is important for the diagnosis and treatment of patients. We formerly had to detect these samples twice by using one assay for bacteria and another for viruses, but now we can obtain the results through one test. This is good news for both patients and doctors. Doctors can obtain the infection statement as soon as possible and prescribe the expected treatment, and patients can save money and obtain the right treatment in a timely manner. Due to the fact that it will take some time to transform the RNA nucleic acid into cDNA for later amplification and signal detection, the limit of detection and processing time will be slightly longer than to detect DNA alone; however, the sensitivity of our system for MV RNA can still reach 1E+2 copies, and we are conducting further research to achieve a sensitivity of 1E+1 copies of RNA templates.

(2) Fast detection with high sensitivity and specificity.

This is our first attempt to combine two kinds of isothermal amplification methods, which merges the advantages of RPA and LAMP, while circumventing their shortcomings. RPA can produce 109 times the products in 30 min, but it has a tendency to produce spurious amplicons from primer-dimer or primer-probe complexes [39]. In fact, we also tried fluorescence RPA (probes see Table 1) in this study; however, we obtained similar false positive results for negative controls, as primers will produce dimers at low temperature. LAMP is highly specific, but its sensitivity is slightly inferior to RPA. By processing a first-stage RPA to reproduce a large number of targets in a short period of time, and a second-stage fluoresce LAMP to detect targets specifically, we can reach a sensitivity of about 10- and 100-fold better than LAMP alone, when operating with unpurified samples and purified RPA products, respectively.

(3) Accurate parallel detection of multiple targets with a small volume.

The microfluidic chip format lays a foundation for the capability of parallel detection (as many as 22 targets) for general pathogens (including DNA and RNA) in around one hour. The risk of contamination for multiple target detection has been minimized for two reasons: (1) products of first-stage RPA can be centrifuged, and diffuse into the second-stage fluorescence LAMP reaction components by mechanical force in a closed system, and (2) all of the LAMP primers have been planted in bioreactors in advance, so there is no need to expose the first-stage RPA products, which are rich in nucleic acid, and LAMP primers, which are rich in primers, to the environment. Moreover, it is cost effective to employ the chip method rather than the traditional tube method. While it consumes 50 μL and 25 μL reagents for RPA and LAMP, respectively, in the tube for each target, the reaction volume on the chip (2.1 μL for RPA and 10.6 μL for LAMP) is approximately 23.8 times and 2.4 times smaller than that in the tube, and thus the consumption of reaction components is largely reduced. In addition, the two-stage isothermal amplification microfluidic chip assay only requires a portable real-time fluorescence detector for reaction and result reporting, which is much more suitable than large-scale PCR amplification instruments in terms of simplicity and convenience.

In summary, the two-stage isothermal amplification method—which consists of a first-stage basic RPA and a second-stage fluorescence LAMP, while implemented in a microfluidic chip format—is capable of detecting multiplex targets (including DNA and RNA) in a short time, with high sensitivity and specificity. The technology and methods in this paper are of value to develop a fully integrated diagnosis system, especially for implementation in POCT.

## Figures and Tables

**Table 1:** Primers for SARS-CoV-2 and MV.

Target	Primer Name	Primer Sequence (5′-3′)
SARS-CoV-2 (CoV-2)	CoV-RPA-F	ATACACTAATTCTTTCACACGTGGTGTTTA
	CoV-RPA-R	AGTAGCGTTATTAACAATAAGTAGGGACTGGG
	CoV-LAMP-F3	ACACTAATTCTTTCACACGTGGTG
	CoV-LAMP-B3	ATTAACAATAAGTAGGGACTGGG
	CoV-LAMP-FIP	CCAGAGACATGTATAGCATGGAACCCATTCAACTCAGGACTTGTTCT
	CoV-LAMP-BIP	GAGGTTTGATAACCCTGTCCTACCATCTTCGAATCTAAAGTAGTACC
	CoV-LAMP-LF	CATTGGAAAAGAAAGGTA
	CoV-LAMP-LB	TGCTTCCACTGAGAAG
Measles Virus (MV)	MV-RPA-F	AGAATAATGAAGAAGGGGGAGACTATTATGA
	MV-RPA-R	CAGCAGCAGCTGTCCTCTGGAACTGGTCCG
	MV-LAMP-F3	GGACACCTCTCAAGCATC
	MV-LAMP-B3	CAGCAGCTGTCCTCTGGAA
	MV-LAMP-FIP	CGGCCTGAATCTCTGCCTATGATTGGGAAGGATCCCAACG
	MV-LAMP-BIP	GTTCTCAAGAAACCCGCTGCCCTGGTCCGTCCATTTGTCA
	MV-LAMP-LF	GGATTGAGTTCGACATCTGC
	MV-LAMP-LB	AGCCGACAACTCCAAGGA

**Table 2:** Tp values and SD of bRPA-LAMP and LAMP alone with 1E+4, 1E+3, 1E+2, and 1E+1 copies of MV RNA as target (*N* = 3). NS stands for non-detectable signal during the duration of the experiment.

Tp ± SD (min, *N* = 3)				
	1E+4 Copies	1E+3 Copies	1E+2 Copies	1E+1 Copies
LAMP	17.8 ± 0.6	22.8 ± 0.9	2 in 3 positive	NS
Unpurified RPA product followed by LAMP	8.4 ± 0.2	11.8 ± 0.2	35.3 ± 1.4	2 in 3 positive
Purified RPA product followed by LAMP	8.3 ± 0.1	10.4 ± 0.1	14.4 ± 0.2	21.3 ± 0.5

**Table 3:** Tp values and SD of bRPA-LAMP and LAMP with concentration of 1E+3, 1E+2, and 1E+1 copies of MV RNA on microfluidic chip (*N* = 3).

Tp ± SD (min, *N* = 3)			
	1E+3 Copies	1E+2 Copies	1E+1 Copies
bRPA-LAMP	12.33 ± 0.08	17.26 ± 0.07	21.02 ± 0.41
LAMP	15.07 ± 0.02	21.77 ± 0.46	1 in 3 positive

**Table 4:** Test of the specificities of the microfluidic chip to SARS-CoV-2 and MV RNA in nasopharyngeal swab samples.

Targets		Culture	On Microfluidic Chip	Clinical Sensitivity	Clinical Specificity
SARS-CoV-2	Positive	17	16	94.12%	95.83%
	Negative	23	24		
MV	Positive	23	23	100.0%	100.0%
	Negative	17	17		
